# Watching synchronous mitochondrial respiration in the retina and its instability in a mouse model of macular degeneration

**DOI:** 10.1038/s41598-021-82811-2

**Published:** 2021-02-08

**Authors:** Pardis Kaynezhad, Ilias Tachtsidis, Asmaa Aboelnour, Sobha Sivaprasad, Glen Jeffery

**Affiliations:** 1 Institute of Ophthalmology, University College London, London, EC1V 9EL UK; 2 Department of Medical Physics and Biomedical Engineering, University College London, London, WC1E 6BT UK; 3 Histology and Cytology Department, Faculty of Veterinary Medicine, Damanhour University, Damanhour, Egypt

**Keywords:** Biophotonics, Optical spectroscopy

## Abstract

Mitochondrial function declines with age and in some diseases, but we have been unable to analyze this in vivo. Here, we optically examine retinal mitochondrial function as well as choroidal oxygenation and hemodynamics in aging C57 and complement factor H (CFH^−/−^) mice, proposed models of macular degeneration which suffer early retinal mitochondrial decline. In young C57s mitochondrial populations respire in coupled oscillatory behavior in cycles of ~ 8 min, which is phase linked to choroidal oscillatory hemodynamics. In aging C57s, the oscillations are less regular being ~ 14 min and more dissociated from choroidal hemodynamics. The mitochondrial oscillatory cycles are extended in CFH^−/−^ mice being ~ 16 min and are further dissociated from choroidal hemodynamics. Mitochondrial decline occurs before age-related changes to choroidal vasculature, hence, is the likely origin of oscillatory disruption in hemodynamics. This technology offers a non-invasive technique to detect early retinal disease and its relationship to blood oxygenation in vivo and in real time.

## Introduction

Mitochondria supply the cell with the energy for metabolic function in the form of adenosine triphosphate (ATP). However, they also produce reactive oxygen species (ROS) when their function declines and this has been linked to the pace of aging and progression of disease, particularly in tissues with high metabolic demand including the retina and brain^1^. These dynamic organelles also have the ability to fuse in response to changing energy demands and to show collective behavior in response to oxidative stress where they synchronize their membrane potentials (ΔΨm) and show clustered oscillatory behavior in response to elevated ROS^2^.

The outer retina has the greatest energy demand in the body, with the greatest density of mitochondria in photoreceptors^3^. It is often regarded as sitting on the edge of hypoxia during normal function and there are important links between hypoxia and ROS that highlight retinal vulnerability, which can be gateways into disease^4^. Age-related macular degeneration (AMD) is a major cause of blindness and associated with reduced mitochondrial function^5^. In 50% of cases there are polymorphisms of complement factor H (CFH) resulting in premature inflammation^6–8^. In murine AMD models (CFH^−/−^ and CFH^−/+^) there is reduced ATP and mitochondrial decline is apparent very early, soon after retinal differentiation. Further, there is abnormal choroidal thinning that likely increases hypoxia^9–11^. This early decline in the murine model reflects features in those with complement polymorphisms, where choroidal inflammation and abnormal choroidal blood flow are present decades before AMD normally manifests and where premature mitochondrial decline has been identified^5,12,13^.

Mitochondrial respiration can be viewed in real time in vivo with non-contact optical technologies based on near-infrared (NIR) illumination that can quantify the mitochondrial cytochrome-*c*-oxidase oxidation changes (Δ[oxCCO]) as well as changes in oxygenated and deoxygenated hemoglobin (Δ[HbO_2_] and Δ [HHb], respectively) in reflected NIR light from the tissue^14,15^. We have recently demonstrated that we can monitor changes in retina mitochondrial respiration and those in oxygenation and hemodynamics through light reflected from the eye in the NIR range^16^.

NIRS has been used to study slow-wave oscillations in cerebral hemodynamics and metabolism^17–21^. These oscillations have also been investigated using a range of techniques including laser Doppler and fMRI^22–25^. They are characterized by their spontaneity and being distinctly different from other known oscillatory phenomena such as heart rate and respiratory cycles. Here we reveal that periodic synchronous mitochondrial oscillations are a key feature of the normal retina in young C57B6 mice but are undermined in early aging and by the CFH^−/−^ genotype resulting in a dissociation of mitochondrial respiration and hemodynamics.

## Results

### Retinal oscillations degrade with age and in CFH^−/−^

**Figure 1:**
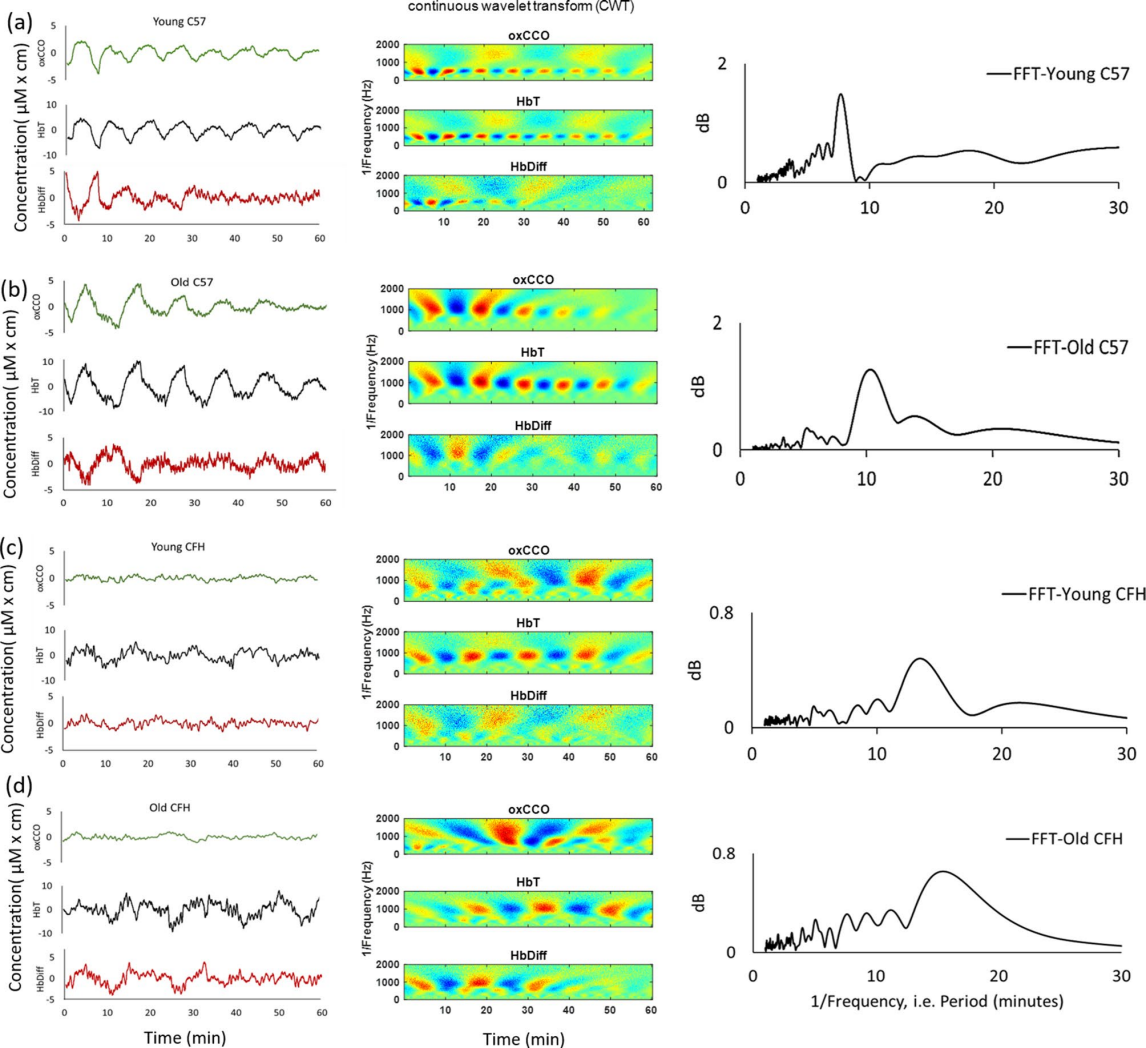
Retinal oscillations are periodic and degrade with age and in CFH^−/−^. Mitochondrial and blood oscillations in a young C57 (**a**), an old C57 (**b**), a young CFH^−/−^ (**c**) and an old CFH^−/−^ mouse (**d**). In the left column green represents oxidized cytochrome-*c*-oxidase (oxCCO), black is total hemoglobin (HbT) which is a marker for blood volume being the sum of oxygenated and deoxygenated hemoglobin (HbO_2_ and HHb, respectively) and the red represents changes in retinal hemoglobin difference (HbDiff = HbO_2_ – HHb) which signifies retinal tissue oxygenation and is sensitive to oxygen delivery. The middle column is complex cross-wavelet transforms (CWT) of the signals in the first column providing the time frequency analysis of mitochondrial (oxCCO) and blood (HbT and HbDiff) signals. The CWT images are produced using a MATLAB program, Comparing Time Series using Semblance Analysis, version 1.0.0.0, found at https://uk.mathworks.com/matlabcentral/fileexchange/18409-comparing-time-series-using-semblance-analysis. Red indicates a positive amplitude and blue a negative. Oscillation regularity in young C57 are revealed with more clearly defined red and blue areas in the CWT images. Mean Fast Fourier Transform of blood and mitochondrial signal oscillations are presented on the right column. The largest peak in the frequency spectrum of each FFT signal provides the most predominant frequency of slow-wave oscillations shown on the left column. These shift to the left running down the column and become less clearly defined.

Spontaneous slow-wave synchronous oscillations with frequencies between 0.001–0.002 Hz, were found in mitochondrial and blood signals, as seen in examples from each group of mice shown in Fig. 1. The left column shows the mitochondrial and blood oscillations. These synchronous oscillations had increased periodicity and reduced regularity with age and CFH^−/−^ genotype. No difference was observed in the amplitude of oscillations of mitochondrial or blood signals in aged or CFH^−/−^ mice. The second column shows the complex cross-wavelet analysis, again showing similar age and genotype differences between mice in the separate groups. Separation between the red and blue colors for each animal reflects increased regularity of oscillations between the signals. Color separation is higher in young C57 than old, and markedly more disrupted in both young and old CFH^−/−^ mice. FFT analysis in the third column revealed that all the oscillations are periodic in each animal example, but this periodicity is more regular in young C57 than old, and progressively less so in the CFH^−/−^ as the peak frequency moves progressively to the right and broadens.

**Figure 2:**
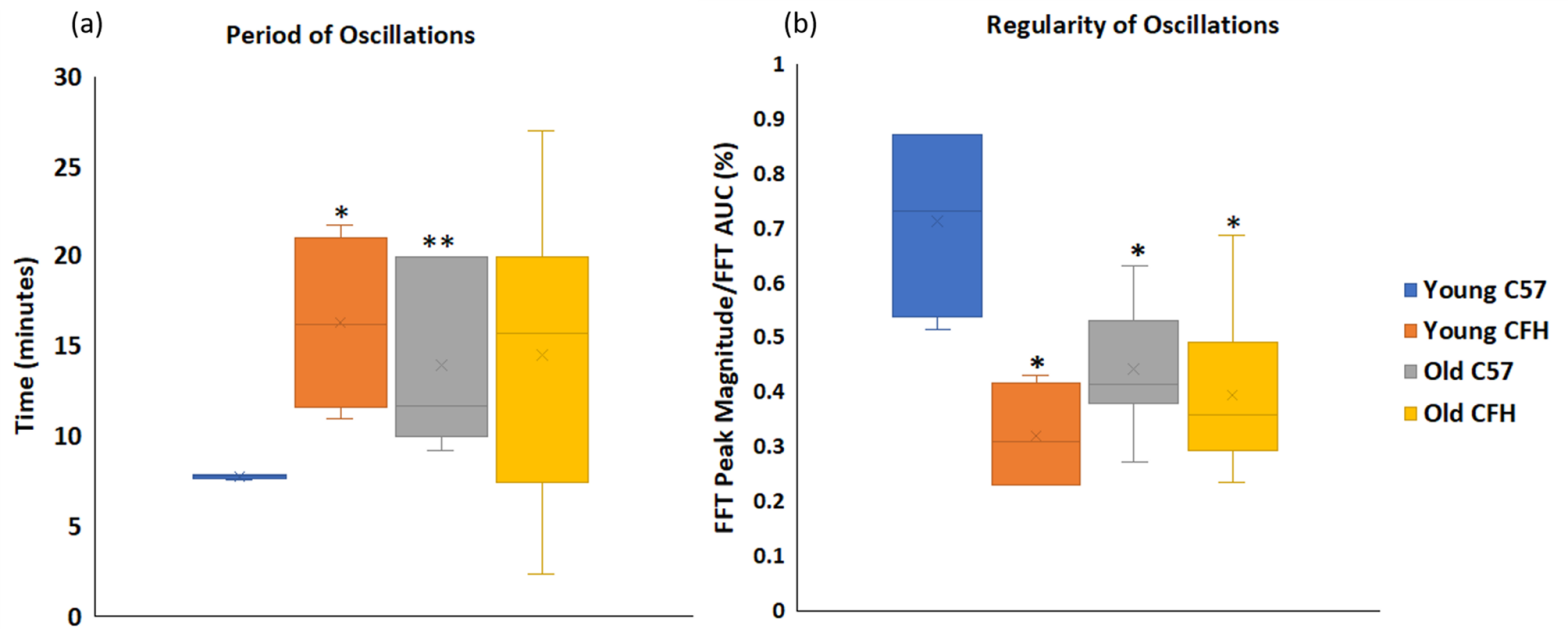
Oscillations in young C57s have higher frequency and regularity than old C57 and CFH^−/−^. The period (**a**) and regularity (**b**) of mitochondrial (oxCCO) and blood (HbT = HHb + HbO_2_ and HbDiff = HbO_2_ − HHb) oscillations in the 4 populations; C57 (n = 4), old C57 (n = 7), young CFH^−/−^ (n = 4) and old CFH^−/−^ (n = 9) mice. (a) Young C57 have the shortest oscillation period (7.8 ± 0.07 min) compared with old C57 (14 ± 1.8 min), young CFH (16.3 ± 2.5 min) and old CFH^−/−^ (16 ± 2.3 min). Oscillations in young C57 retinae have a 2 MHz frequency which is greater than that in other groups. (**b**) Shows that oscillations have the highest regularity in young C57 mice (0.7 ± 0.1 (%). Regularity is defined as the magnitude of peak Fast Fourier Transform (FFT) divided by the Area Under the Curve (AUC) of the frequency spectrum). Data are mean ± standard error (*p < 0.05, **p < 0.005).

The group population data for periodicity and regularity are given in Fig. 2. The young C57 mice stand separately from other groups in both metrics. The young C57 had the smallest oscillation period for mitochondrial and blood signals (8 ± 0.07 min), compared with the old C57 (14 ± 1.8 min, p = 0.006), young CFH^−/−^ (16 ± 2.5 min, p = 0.03) and old CFH^−/−^ mice (16 ± 2.3 min, p = 0.1) as seen in Fig. 2a. Mitochondrial and blood oscillations in young C57 mice were also statistically more regular than other groups (Fig. 2b). The regularity index for oscillations in young C57s, quantified through dividing the peak FFT magnitude by the area under the curve of the FFT signal, was 0.7 ± 0.1%, which signifies a high degree of periodicity and it was significantly greater than that for other groups. In old C57 mice, the regularity index dropped to 0.43 ± 0.1%, p = 0.03; and it was reduced further in young and old CFH^−/−^ being 0.3 ± 0.06%, p = 0.04 and 0.39 ± 0.05%, p = 0.02, respectively.

**Figure 3:**
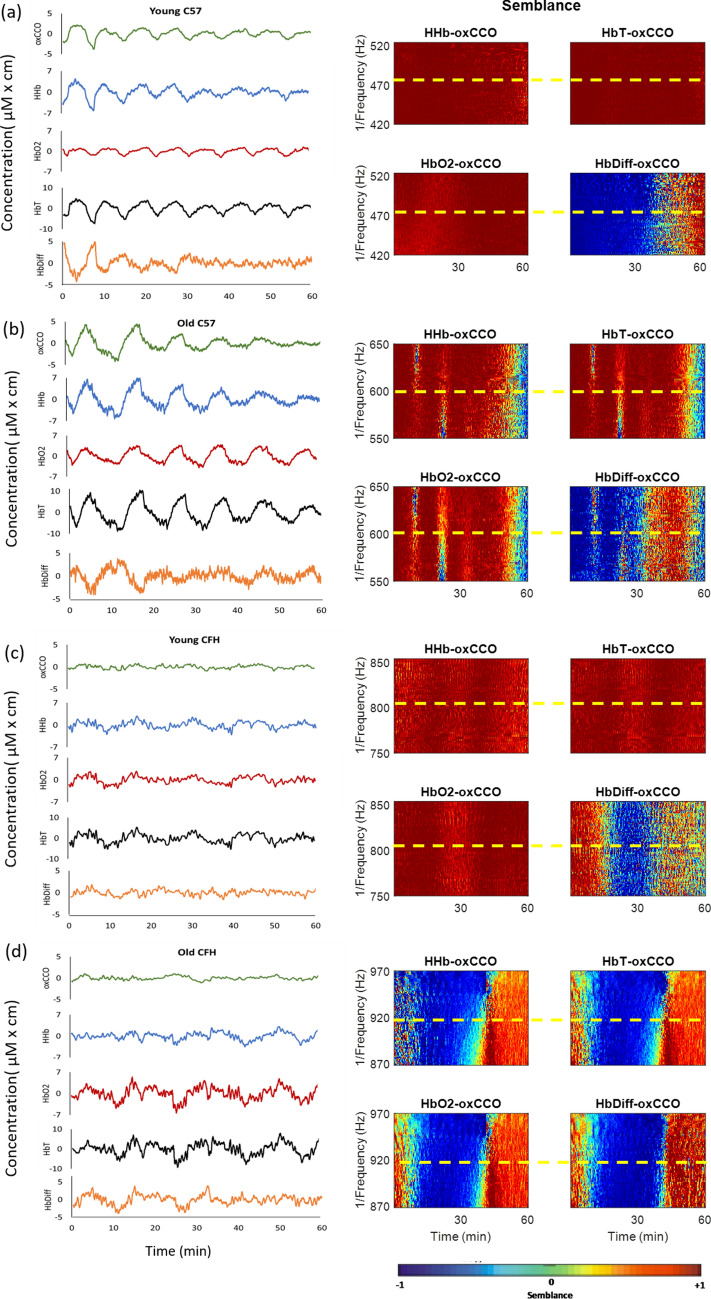
The strong phase link between mitochondrial and choroidal oscillations in young C57 mice is degraded with age and in CFH^−/−^. Instantaneous phase difference (wavelet semblance) between oxidation of cytochrome-*c*-oxidase (oxCCO) with deoxygenated and oxygenated hemoglobin (HHb and HbO_2_). These data are presented along with total and difference hemoglobin (HbT and HbDiff, measures of total blood volume and oxygen delivery) in examples of each group of mice. The left column is the slow-wave oscillations in mitochondria and blood signals over 1 h [young C57 (**a**), old C57 (**b**), young CFH^−/−^ (**c**), old CFH^−/−^ (**d**)], followed by their corresponding semblance color maps on the second and third columns. The color maps (Hb-oxCCO semblance) represent phase relationship between oxCCO and Hb signals at different frequencies (y-axis) over time (x-axis). The color maps are produced using a MATLAB program, Comparing Time Series using Semblance Analysis, version 1.0.0.0, found at https://uk.mathworks.com/matlabcentral/fileexchange/18409-comparing-time-series-using-semblance-analysis. To demonstrate the relationship between mitochondria and blood signals during the slow-wave oscillations, we have selectively magnified the y-axis for each mouse and set the limits to 1/ slow-wave oscillation frequency ± 50 s. The horizontal dotted line corresponds to the specific oscillation frequency for each mouse. Areas in red color represent a semblance of + 1 implying perfect phase correlation (zero-degree phase difference), green corresponds to a semblance of zero signifying no phase correlation (90° phase difference), and dark blue indicates perfect anticorrelation (180° phase difference) corresponding to a semblance of − 1.

Figure 3 presents examples of semblance color images between mitochondria and blood signals from mice in each group. A semblance of + 1, signifying a zero-phase difference is shown in red, and a semblance of − 1, which shows a 180° phase difference is demonstrated in blue. The left column shows all the NIRS signals over 1 h and the next two columns represent the semblance between mitochondrial (oxCCO) and blood signals during slow-wave oscillations specific to each mouse over time. The y-axis limit is set based on the 1/oscillation frequency for each mouse ± 50 s. The horizontal dotted line represents phase relationship between mitochondria and each blood signal at the specific oscillation frequency for that animal. The semblance between oxCCO with deoxygenated and oxygenated hemoglobin signals (HHb and HbO_2_) are presented in the second column and the semblance between oxCCO with total and difference hemoglobin signals (HbT and HbDiff), which are measures for total retinal blood volume and retinal oxygenation, are presented in the third column.

In the examples, mitochondrial oscillations are perfectly in phase with HHb, HbO_2_ and HbT oscillations in the young C57 mouse (first row), represented in a consistently red color over time, at the oscillation frequency. The oscillations of HbDiff signal however, are perfectly antiphase with oxCCO oscillations (a consistently blue color) and change over time. In the old C57 mouse, shown on the second row, oxCCO oscillations are in phase with HHb, HbO_2_ and HbT with minor disturbance and antiphase with HbDiff, changing semblance over time. Likewise, mitochondrial oscillations in the young CFH^−/−^ mouse shown on the third row are in phase with oxy-, deoxy- and total hemoglobin signals and change phase with the HbDiff signal. Finally, the semblance color maps for mitochondrial-blood signals in the old CFH^−/−^ mouse shown on the last row, reveal significant inconsistency in phase association between mitochondria and all the blood signals during measurement. In these examples, oxCCO semblance with HHb, HbO_2_ and HbT is almost completely maintained in the C57 across the two ages and in the young CFH^−/−^ mouse, but shows marked disruption in the old CFH^−/−^.

**Figure 4:**
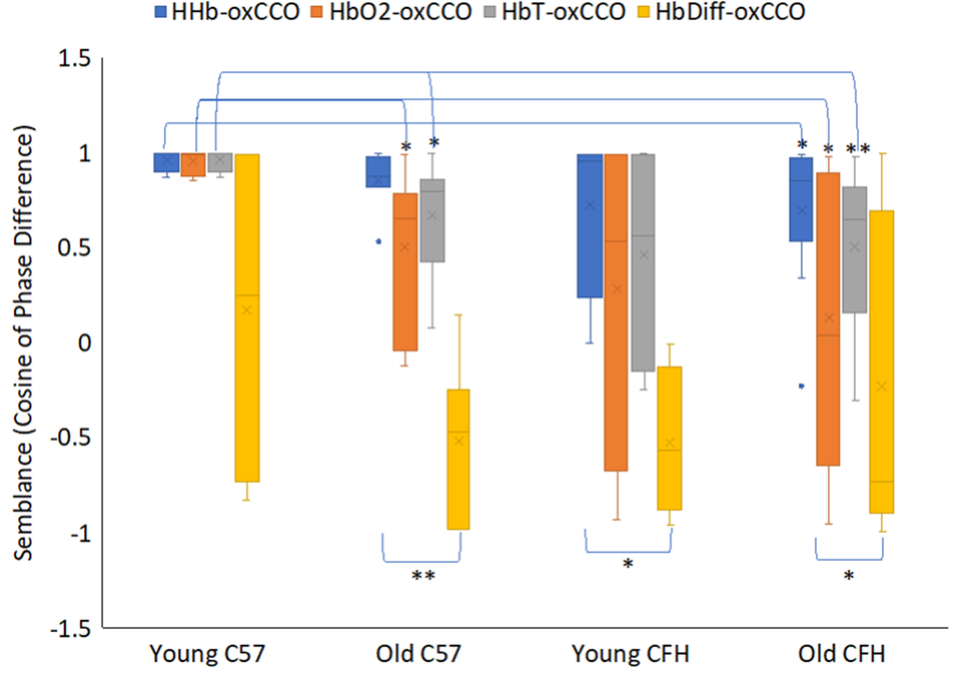
The association between mitochondrial and choroidal signals during slow-wave oscillations is significantly reduced with age and in CFH^−/−^. Oscillatory phase differences (wavelet semblance) between hemoglobin (HHb, HbO_2_, HbT and HbDiff) and oxCCO signals in different experimental groups averaged over 1 h. The mean semblance for each animal is calculated at the specific slow-wave oscillation frequency for that animal (the most predominant peak on the FFT signal). A semblance of + 1 indicates that the hemoglobin signal oscillations are perfectly in phase with the oscillations in the oxCCO signal, and − 1 that they are perfectly antiphase with oxCCO oscillations. In young C57 mice (n = 4), oscillations in oxygenated and deoxygenated hemoglobin (HbO_2_ and HHb) and total hemoglobin (HbT) are in phase with mitochondrial oscillation (oxCCO). The phase difference between oxygenated and total hemoglobin (HbO_2_ and HbT) with metabolism (oxCCO) are significantly greater in old C57 (n = 7) and old CFH^−/−^ (n = 9) mice compared to young C57 mice, but not in young CFH^−/−^ mice (n = 4). In all groups HHb oscillations follow the mitochondrial oscillations more closely than other hemoglobin signals, and except for young C57s, there is a significant difference between the semblances of HHb-oxCCO with HbDiff-oxCCO. Asterisks are significant differences from HbDiff-oxCCO semblance. Data are mean ± standard error (*p < 0.05, **p < 0.005, ****p < 0.00005, *****p < 0.00001).

Group analysis for the semblance values between mitochondrial and blood signals for the metrics in Fig. 3 are shown in Fig. 4. Semblance is the cosine of the phase difference, and a + 1 semblance means that two signals are in perfect phase. Here in young C57 mice the oscillations of HbO_2_, HHb and HbT signals are almost perfectly in phase with mitochondrial oscillation (oxCCO) with semblance values of around + 1. There is a significantly greater phase difference between oscillations of oxygenated blood and total blood volume (HbO_2_ and HbT) with oxCCO in old C57 and old CFH^−/−^ mice compared to young C57 mice. In all groups, HHb signal oscillations follow the mitochondrial oscillations more closely, having smallest phase differences (highest semblance) compared to other hemoglobin signals, and except for the young C57s, the semblance of HbDiff with oxCCO is significantly smaller than the semblance of HHb with oxCCO during oscillations. HHb signal oscillations correspond to periodic changes in the concentration of deoxygenated blood in the retinal tissue. The fact that variations in the concentration of deoxygenated blood closely follows changes in the concentration of oxidized cytochrome-*c*-oxidase implies periodic increase/decrease in oxygen consumption. As consumption increases, there is a need for increased oxygenation (HbDiff) and there is a significantly large phase delay between HbDiff-oxCCO compared to HHb-oxCCO in old C57, young CFH^−/−^ and old CFH^−/−^ mice. Hence, with age and in CFH^−/−^ mice there is a large delay between oxygen supply and demand during oscillations.

**Figure 5:**
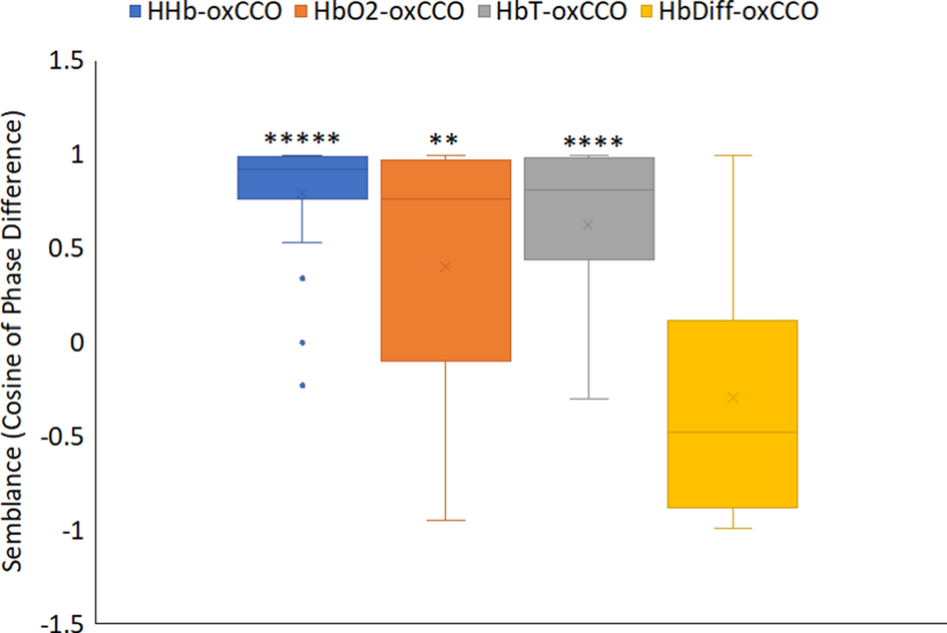
Oscillations in choroidal hemoglobin difference (blood oxygenation, HbDiff) has the greatest dissociation (smallest semblance) with oscillations in retinal Metabolism. Oscillatory phase differences (wavelet semblance) between hemoglobin (HHb, HbO_2_, HbT and HbDiff) and oxCCO signals in all the mice (n = 24) averaged over 1 h. The mean semblance for each animal is calculated at the specific slow-wave oscillation frequency for that animal (the most predominant peak on the FFT signal. Here, there is a significantly greater phase difference between mitochondrial metabolism (oxCCO) with hemoglobin oxygenation (HbDiff) corresponding to oxygen delivery. Semblance of + 1 implies that hemoglobin signal oscillations are in perfect phase with oscillations in the oxCCO signal. Semblance of − 1 implies that they are perfectly anticorrelated. The semblance of HHb, HbO_2_ and HbT with oxCCO signals in all the mice are significantly greater than the semblance of HbDiff-oxCCO as compared using Wilcoxon Rank sum test. Data are mean ± standard error and the statistical significance is for each group against the right hand boxplot (HbDiff-oxCCO semblance) (**p < 0.005, ****p < 0.00005, *****p < 0.00001).

The phase difference between mitochondrial and blood oscillation in all mice was examined (Fig. 5). Here the semblance between HbDiff and oxCCO oscillations (HbDiff-oxCCO = − 0.3 ± 0.1) was significantly smaller than that of oscillations of other hemoglobin signals with oxCCO (HHb-oxCCO = 0.8 ± 0.07, p = 0.000008, HbO_2_-oxCCO = 0.4 ± 0.1, p = 0.003 and HbT-oxCCO = 0.6 ± 0.08, p = 0.00004). Hence, in all mice regardless of age or genotype, there is a significantly greater phase difference between mitochondrial (oxCCO) oscillations with oscillations in retinal oxygenation (HbDiff). Knowing that the HbDiff signal is sensitive to oxygen delivery^26–28^ may indicate that the large phase difference between the oscillations in oxygenation and metabolism reflects that mitochondrial oscillations are not a direct response to increase or decrease in oxygen delivery and have a metabolic source.

### Retinal oscillations originate from mitochondria

**Figure 6:**
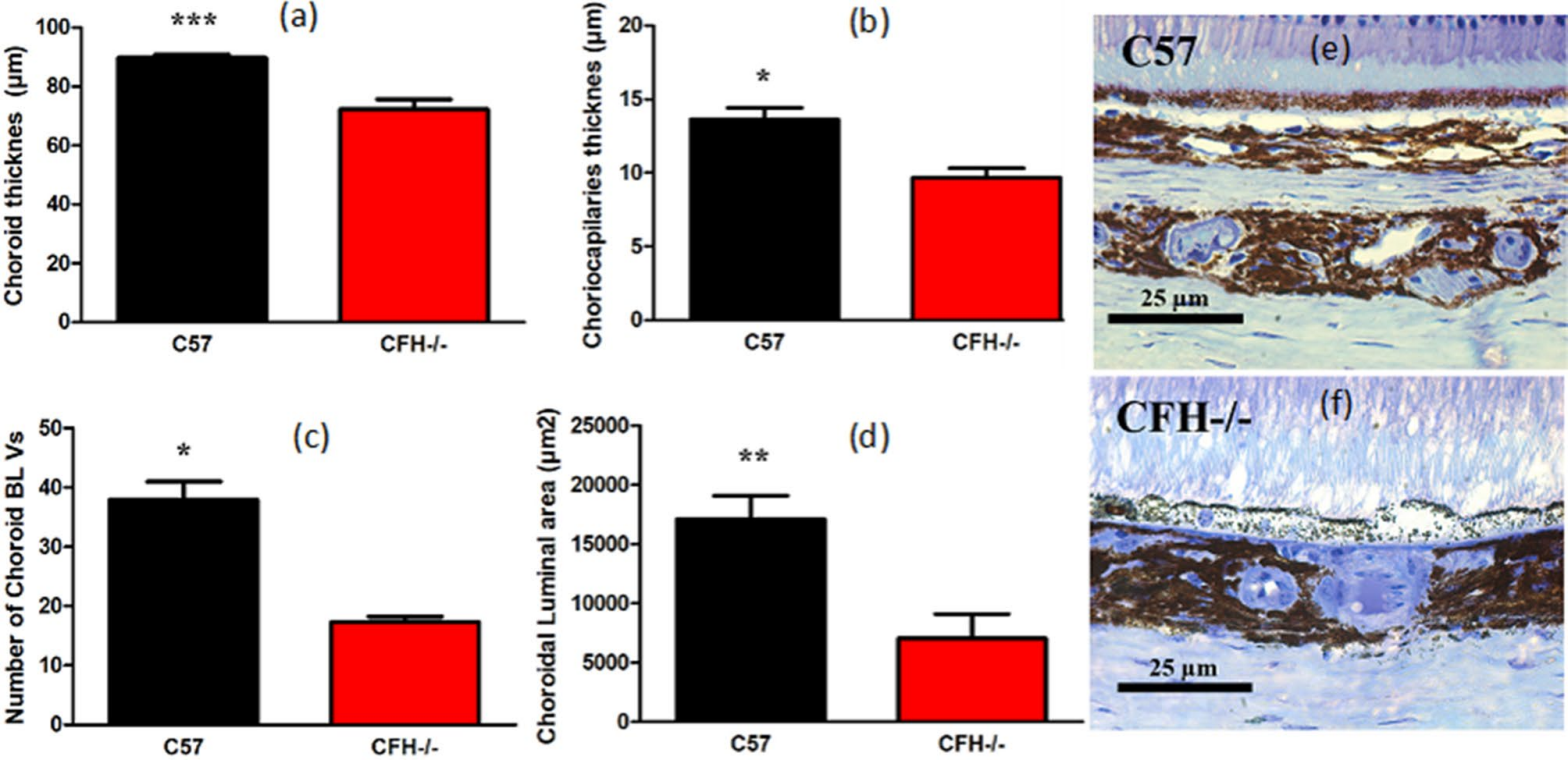
Measurements of choroidal integrity in C57 and CFH^−/−^ mice at 6 months of age, produced using Adobe Photoshop Cs5 extended software, version 12.0. × 64. The choroid in CFH^−/−^ mice (n = 4) is significantly reduced compared to the C57 (n = 4). This is true for total choroidal thickness (**a**), the thickness of individual choriocapillaris (**b**), the number of choroidal blood vessels (**c**) and the choroidal luminal area (**d**). (**e**,**f**) are respectively histological examples from C57 and CFH^−/−^ mice. The choroid is the bottom just above the scale bar. The neural retina is to the top. Data are mean ± standard error (BL vs, Blood vessels, *p < 0.05, **p < 0.005, ***p < 0.00005).

Choroidal thickness has previously been examined in C57 and CFH^−/−^. In CFH^−/−^ it is normal at 3 months but reduced by > 50% at 12 months compared to C57^9^. Hence, here we examined the choroid at 6 months (Fig. 6) and found a significant reduction in choroidal thickness of approximately 20% and a significant reduction in the number of choroidal blood vessels of > 50% in CFH^−/−^, suggestive of reduced outer retinal perfusion. However, despite this, we found no difference in periodicity or regularity of oscillations between old C57 and old CFH^−/−^ (Fig. 2). Based on the wavelet semblance analysis, neither did we observe any differences in phase association between all the hemoglobin signals with metabolism during oscillations between old C57 and old CFH^−/−^ mice. This strongly suggests that the slow-wave retinal oscillations have a mitochondrial origin rather than one associated with choroidal flow.

## Discussion

This study demonstrates synchronized self-sustaining periodic oscillatory behavior in retinal mitochondria and oxygenation/hemodynamics using non-invasive in-vivo optical measurements in real time. This feature of mitochondrial populations has not been seen previously in healthy tissues. However, the retina has the highest metabolic rate in the body with the greatest density of mitochondria in photoreceptor inner segments and is a naturally stressed environment^3,29^. Perhaps synchronized respiration may be a strategy for coping with metabolic stress similar to that found in hypoxic cardiac tissue where synchronous mitochondrial oscillatory behavior is initiated and modulated by elevated ROS^2,30^. While this is a distinct feature, the underlying mechanisms are far from clear, although the changes that occur with age and genotype signify their importance.

The slow-wave synchronous retinal oscillations were periodic as revealed by FFT analysis. In young C57 mice they were at approximately 8 min intervals but were significantly longer and less regular in older C57 and in CFH^−/−^ mice. Oscillation frequency varied between 0.001–0.002 Hz, which is notably smaller than the frequency of previously reported physiological oscillations in mitochondria or hemodynamics^2,18,20,21,31^. Cerebral slow-wave oscillations in oxy- and deoxy-hemoglobin reported in the literature are categorized into two groups of low frequency (0.07–0.11 Hz) and very low frequency (0.01–0.05 Hz) and they are mainly associated with cerebral autoregulation^18,21^.

Mitochondrial and hemodynamic signals were correlated with instantaneous phase relationships between them measured by wavelet semblance analysis. This demonstrated that in young C57 mice mitochondrial oscillations were in phase with oscillations in oxygenated and deoxygenated blood, but this phase relationship was weaker in other groups, although this was not always statistically significant. Changes in deoxygenated blood is closely linked to changes in oxidation of cytochrome-*c*-oxidase during oscillations in all animals, having the highest mean wavelet semblance with oxCCO compared to other hemoglobin signals. The small phase delay between HHb and oxCCO signals during oscillations implies periodic shifts in tissue oxygen consumption. Cytochrome-*c*-oxidase as the terminal enzyme in the electron transport chain of mitochondria is oxidized periodically, which is simultaneous with increase/decrease in retinal deoxygenated blood and indicates enhanced/inhibited aerobic metabolism.

Oscillations in the oxygenated blood had a greater phase difference with oxCCO compared with deoxygenated blood in all animals, except in young C57 mice, implying that there is an imbalance between oxygen demand with choroidal supply. There is a significant loss of phase synchrony between HbO_2_ and oxCCO signals in older C57 mice compared to young ones, which may be due to an aging choroid. In CFH^−/−^ mice, the choroid is normal at 3 months^9^, while mitochondrial abnormalities are present from 3 weeks with a significant reduction in ATP at 4 months^10,11^. Therefore, the notably lower frequency and reduced oscillation regularity in young CFH^−/−^ mice are likely to have a mitochondrial origin rather than one of blood supply. CFH is predominantly expressed in the liver and RPE^32^ and its absence in the RPE is associated with premature inflammation. As the RPE sits adjacent to the choroid, inflammation here initiated between 4–6 months^33^ may relate to choroidal collapse. This could explain why there is not a significant loss of phase synchrony between HbO_2_ and oxCCO signals in young CFH^−/−^ mice.

In all animals, oscillations in retinal blood oxygenation (HbDiff), which is sensitive to oxygen delivery^26–28^, had a significantly lower wavelet semblance with mitochondrial oscillations (oxCCO) compared to other hemoglobin signals. The large phase delay or dissociation between HbDiff and oxCCO signals during oscillations implies that periodic shifts in mitochondrial oxidative metabolism are not a direct response to enhanced or reduced oxygen delivery or blood flow but may be intrinsic.

A key question for our study is the origin of the signals we measure. The mitochondrial source most likely originates from photoreceptor inner segments as they contain the greatest mitochondrial density in the body and this region contains little else^3^. There are two potential sources for our blood signals, the retinal and the choroidal circulation. The choroidal circulation is by far the larger, supplying the outer retina with a relatively uniform distribution. The smaller retinal circulation is supplied by radially distributed vessels originating at the optic nerve head. The phase locking of mitochondrial signals with those of the blood implies that our blood signals were largely choroidal. Although this sits behind RPE, its pigmentation is not a significant barrier to the long wavelength signals that were detected. The RPE is relatively transparent to the wavelengths we use to resolve concentrations of HHb, HbO_2_ and oxCCO (780–900 nm). Hence, it is most likely that a significant proportion of signal arise from the choroid. Other studies have also used similar wavelengths to penetrate the RPE and image the choroid^34,35^. Further, we did not restrain our animals and small eye movements did occur. Had our blood signals originated predominantly from the radial retinal circulation we may have expected eye movements to result in changes to the vessels sampled within recording regions, resulting in changes in signal strength. But this did not occur. Hence, we argue that our mitochondrial and blood signals were linked in the outer retina.

We used a low power halogen-based white light source to illuminate the eye and resulting reflections were monitored for 1 h. Even though the total power transferred was < 6 mW, we cannot completely exclude the possibility that oscillations were a response to light exposure. However, oscillations were present when recordings were initiated, suggesting that they are inherent. Further, we used a range of light intensities greater than a log unit and some of these were dim to the human eye. In spite of this, we found no relationship between light intensity and the pattern of oscillations. This has also been concluded in other mitochondrial studies, where different excitation wavelengths and energies were used, suggesting that photooxidation plays a minor role in pulsing compared with biological effects^36^. An additional consideration is that the retina is under elevated metabolic stress in darkness due to maintenance of the dark current^3^. Hence, our illumination, particularly at lower luminance was unlikely to be detrimental. Taken together, it is likely that the oscillations we reveal are a natural mechanism for coping in the stressed retinal environment.

Membrane fluctuations have been reported in isolated mitochondria from brain, liver and in plants, although explanations for this lack consistency^37–41^. However, Schwarzlander et al. proposed a model to describe pulsing plant mitochondrial membrane potential, showing that they are triggered by ROS and arguing that they provide quality control and maintenance of mitochondrial function, which in turn reduces ROS production^36^. This is consistent with research on cardiac tissue where synchronous mitochondrial oscillations were increased by ROS^30^. Others have also reported high frequency (~ 50 ms) metabolic spontaneous oscillations in leukocytes using flavoprotein autofluorescence imaging^42^.

How might mitochondria in different cells communicate? Recently, it has been shown that there is a close spatial association between some mitochondria and the plasma membrane in mouse photoreceptor inner segments (IS). Further, some display alignment with their neighbors in adjacent cells across the membranes and there are visible tethers between them at relatively consistent distances that appear to connect them even though they are in different cells^43^. This may suggest trans-cellular mitochondrial communication that could be the structural basis for the synchronized oscillations we reveal here.

It is not known if the degradation of mitochondrial oscillations in CFH^−/−^ mice is a consequence of their spatial disruption limiting trans-cellular communication or simply due to compromised function. However, at 3 weeks of age there is no obvious difference in mitochondrial positioning along the IS plasma membrane between CFH^−/−^ and C57 mice. But mitochondria in CFH^−/−^ mice were significantly enlarged consistent with early senescence^11^. Hence, it is likely that degraded oscillations result from reduced mitochondrial function and not positional defects.

We highlight the importance of mitochondrial and hemodynamic oscillations in the healthy retina and how these are degraded by only few months of aging and by the CFH^−/−^ genotype. Greater degradations may be expected with progressive aging, although aged changes in the mouse optics limited further investigation.

Many questions are raised by our findings. All of our experiments were undertaken early in the day, but given the major shifts in mitochondrial function over 24 h^45^, it may be possible that these are reflected in some aspects of oscillatory behavior, but this remains to be explored. Likewise, the high metabolic cost of the photoreceptor dark current at night may again result in oscillatory changes. However, significant traction may be gained in understanding the mechanisms of retinal disease when associated with mitochondrial decline or choroidal compromise. AMD aside, our technologies may reveal mechanisms in diabetic retinopathy and mitochondrial and degenerative diseases of the retina. Retinal metabolic defects have been previously investigated using flavoprotein fluorescent imaging, in which increased flavoprotein autofluorescence correlated with retinal cell dysfunction in aging and some diseases known to be mediated with oxidative stress^44^. Our technique, however, is unique in terms of enabling real-time measurement of metabolism as well as blood oxygenation and hemodynamics. The combined imaging of mitochondrial function with choroidal hemodynamics demonstrated here is likely to cast light on significant process in these events. Scaling up for the human eye would avoid many of the complications of the small mouse eye. Further, current development of our technologies is allowing much shorter real-time assessment of the metrics we investigate, in absolute rather than relative values.

## Methods

The study was carried out in compliance with the ARRIVE guidelines. All animals were used with University College London ethics committee approval and under a UK Home Office project license (PPL 70/8379). All procedures conformed to the United Kingdom Animal License Act (1986) and local regulations. Throughout animals were anaesthetized with 0.3 ml of a mixture of ketamine (0.56 ml) and Dormitor (0.37 ml) in water (0.56 ml) and their pupils dilated with Tropicamide.

For retinal reflection measurements 4 groups of mice were used in this study; young C57 (3 months n = 4), old C57 (6 months n = 7), young CFH^−/−^ (3 months n = 4), old CFH^−/−^ (6 months n = 9). Our choice of ages was determined by the need for complete optical clarity of the eye which we find declines after 6 months. Further, at this age there is already mitochondrial decline in the C57 mouse.

For retinal histology, to examine changes in the choroid, 6 months old animals (4 C57 and 4 CFH^−/−^) were terminally anesthetized and their eyes removed and placed in a mixture of 2% paraformaldehyde and 2% glutaraldehyde in phosphate buffer saline (PBS). These were left in fixative for approximately 2 days at 4°C then washed in PBS. The anterior tissues were then removed to leave an eye cup from one eye from each animal. These were dehydrated and embedded in Technovit 7100 historesin solution (Taab Laboratories Equipment UK). Resin sections were then cut at 5 µm and a serial series collected in central retinal regions. These were mounted on glass slides and stained with 1% Toludine Blue and mounted in Depex mounting medium and coverslipped.

Choroidal measurements were performed using Adobe Photoshop Cs5 extended software, version 12.0. × 64 and obtained in 20 sections at × 10 (microscope and camera) close to the optic nerve using the line tool. Choroidal thickness was defined as the distance between Bruch’s membrane (the lower boundary of retinal pigmented epithelium (RPE) and the choroid-sclera interface. Choriocapillaris thickness was defined as the distance between Bruch’s membrane and the upper boundary of the stroma. The luminal areas of the choroidal vessels, choroid and choriocapillaris were measured using lasso area tool, providing value for the enclosed vessel luminal areas and the stromal area. The number of choroid vessels was counted manually in the whole length of the stromal area measured.

### Broadband near infrared spectroscopy

Mitochondrial activity in the retinae of mice were measured in real time using a miniature broadband near-infrared spectroscopy (bNIRS) system called miniCYRIL (Cytochrome Research Instrument and application) described previously^16,46^. miniCYRIL consists of a miniature halogen-based white light source (HL2000-HP) and a miniature spectrometer (QE65 pro) by Ocean Optics, USA. Two customized optical fibers (LOPTEK, Germany) transmitted light to the retina and collected the reflected light.

The experiments were performed in darkness and they were generally initiated in the morning and early afternoon. We did not see time of day as a significant variable in these experiments. The animals were lightly anesthetized with 6% ketamine and 10% dormitor (National Veterinary Services UK) and 84% H_2_O at 5 µ/g. Pupils were dilated with 1% Tropicamide (Bausch and Lomb, France). The cornea was lubricated with Viscotears (Novartis, Switzerland). Animals were placed on their sides unrestrained in a temperature controlled clear Perspex cabinet through which optical fibers could be inserted. An antireflective clear glass coverslip was placed on the cornea in contact with the Viscotears to facilitate a clear optical pathway for the optical fibers to deliver light to the retina and collect reflected light back.

The retina was widely illuminated with white light with a total power of < 6 mW without any thermal damage. The source and detector optical fibers were mounted on stereotaxic probe holders in the cabinet at a ~ 40° angle to each other and each placed approximately perpendicular to the corneal surface to avoid specular reflection from the eye.

miniCYRIL converted reflected light from the retina to changes in chromophores’ concentration using the UCLn algorithm based on the modified Beer–Lambert Law^47^. miniCYRIL provides measurements of tissue oxygenation and hemodynamics through measuring changes in oxygenated and deoxygenated hemoglobin (Δ[HbO_2_] and Δ[HHb]) as well as metabolism (through measuring changes in the redox state of cytochrome-*c*-oxidase (Δ[oxCCO]). CCO is the terminal enzyme in the mitochondrial electron transport chain (ETC) and one of its four active metal redox centers, CuA, is a strong NIR absorber with a broad spectral signature. The concentration of cytochrome-*c*-oxidase is one order of magnitude smaller than that of hemoglobin, hence making its in-vivo measurement challenging (15). Use of > 100 wavelengths in bNIRS to resolve the spectral changes due to oxCCO enhances the measurement accuracy without hemoglobin chromophores crosstalk^48^.

The oxCCO measurement has been used in our previous animals and human studies as a reliable marker for cellular oxidative metabolism^31,46,49–52^. Changes in total hemoglobin (Δ[HbT] = Δ[HHb] + Δ[HbO_2_]) as a measure for retinal blood volume as well as hemoglobin difference (Δ[HbDiff] = Δ[HbO_2_] − Δ[HHb]), which is indicative of retinal oxygenation, were also derived.

All animals were monitored for 1 h and the baseline changes in [oxCCO], [HHb], [HbO_2_], [HbT] and [HbDiff] were recorded at a sampling rate of 1 Hz.

### Data processing

Data were analyzed in MATLAB 2019b. All signals were low pass filtered to remove the high frequency oscillations (using a simple lowpass function with cut off frequency 0.01 Hz) and the inevitable large drifts over the measurement period were removed through subtracting a 6th order polynomial fit from all the concentration signals. Fast Fourier transform (FFT) analysis was undertaken to determine the oscillations’ periodicity and regularity quantified through dividing the average peak FFT magnitude by area under the curve (AUC) of the FFT signal. Group data are presented in mean ± SDE (standard error) and non-parametric Wilcoxon rank-sum test was used to compare the period and regularity of oscillations between groups.

To determine phase differences between the dynamic mitochondrial (oxCCO) and blood signals (HHb, HbO_2_, HbT and HbDiff), wavelet-based semblance analysis was undertaken. This method of phase difference analysis is suitable for studying signals with changing spectral and frequency characteristics that vary over time. Briefly, wavelet semblance is the cosine of the instantaneous phase difference between two signals and enables the local phase relationship between them to be studied as a function of both scale (frequency) and time^53^. Semblance varies between + 1, when signals are perfectly in phase, and − 1, when they are antiphase. In this study, semblance between the oscillations in mitochondria (oxCCO) and blood (HHb, HbO_2_, HbT and HbDiff) signals were calculated using continuous wavelet transform (CWT) with the complex Morlet wavelet within the range of slow-wave oscillations of the retina (0.001–0.002 Hz). Semblance analysis was performed using a MATLAB function created by Cooper and Cowan^54^. The function was modified to produce images within the frequency band of slow-wave oscillations specific to each mouse, which was determined through FFT analysis. The mean semblance values between oxCCO and hemoglobin signals were calculated at the most predominant oscillation frequency (peak of the FFT signal) for each mouse. The mean semblance values were then averaged over the 1-h measurement period for comparison using non-parametric Wilcoxon Rank sum test. Statistical significance was considered as p < 0.05.
